# PGRMC1 Inhibits Progesterone-Evoked Proliferation and Ca^2+^ Entry Via STIM2 in MDA-MB-231 Cells

**DOI:** 10.3390/ijms21207641

**Published:** 2020-10-15

**Authors:** Carlos Cantonero, Ginés M. Salido, Juan A. Rosado, Pedro C. Redondo

**Affiliations:** Department of Physiology (PHYCELL Group) of Veterinary Faculty and Institute of Molecular Pathology Biomarkers (IMPB) of University of Extremadura, 10003 Caceres, Spain; carloscantonero@unex.es (C.C.); gsalido@unex.es (G.M.S.); jarosado@unex.es (J.A.R.)

**Keywords:** MDA-MB-231 cells, STIM2, PGRMC1, Ca^2+^-homeostasis, progesterone

## Abstract

Progesterone receptor membrane component 1 (PGRMC1) has been shown to regulate some cancer hallmarks. Progesterone (P_4_) evokes intracellular calcium (Ca^2+^) changes in the triple-negative breast cancer cell lines (MDA-MB-231, MDA-MB-468, and BT-20) and in other breast cancer cell lines like the luminal MCF7 cells. PGRMC1 expression is elevated in MDA-MB-231 and MCF7 cells as compared to non-tumoral MCF10A cell line, and PGRMC1 silencing enhances P_4_-evoked Ca^2+^ mobilization. Here, we found a new P_4_-dependent Ca^2+^ mobilization pathway in MDA-MB-231 cells and other triple-negative breast cancer cells, as well as in MCF7 cells that involved Stromal interaction molecule 2 (STIM2), Calcium release-activated calcium channel protein 1 (Orai1), and Transient Receptor Potential Channel 1 (TRPC1). Stromal interaction molecule 1 (STIM1) was not involved in this novel Ca^2+^ pathway, as evidenced by using siRNA STIM1. PGRMC1 silencing reduced the negative effect of P_4_ on cell proliferation and cell death in MDA-MB-231 cells. In line with the latter observation, Nuclear Factor of Activated T-Cells 1 (NFAT1) nuclear accumulation due to P_4_ incubation for 48 h was enhanced in cells transfected with the small hairpin siRNA against PGRMC1 (shPGRMC1). These results provide evidence for a novel P_4_-evoked Ca^2+^ entry pathway that is downregulated by PGRMC1.

## 1. Introduction

The expression of nuclear progesterone receptor (nPR) is needed for cell proliferation and maturation of breast tissue, and further, nPR expression is found under the regulation of the estrogens [1]. Interestingly, activation of alveologenesis and, subsequently, milk production relies on the balance between progesterone receptor and prolactin/Stat5 signaling [1,2]. Reduction of circulating progesterone (P_4_) concentration facilitates gene expression of the prolactin receptor as well as the activation of its downstream effector, the signal transducer and activator of transcription complex 5 (STAT5) [1,2,3].

Physicians have used the lack or presence of the classical nuclear estrogen receptor (nER) or nPR, in order to histologically characterize the different breast cancer subtypes [4]. Thus, *ESR1*, *PGR,* and *ERBB2* mRNAs are not detected in most of the basal tumor samples and, therefore, also known as triple-negative breast cancer subtype [5]. 

Regarding the role of P_4_ in cell proliferation, it has been reported that P_4_ impairs normal breast epithelial cell proliferation in vivo, as well as in some types of breast cancer cells in vitro [6]. The effect of P_4_ on cell proliferation has been described depending on nPR activation; thus, the triple-negative breast cancer (TNBC) cell lines should remain unaltered in the presence of P_4_; however, recent studies have claimed that P_4_ evokes alteration of the TNBC cells [7,8]. These negative effects in MDA-MB-231 cells are suggested to be mediated by membrane progesterone α (mPRα) [7], which might be modulated by progesterone receptor membrane component 1 (PGRMC1) [1]. Nonetheless, the evidence against mPRα as the P_4_ receptor has also been reported by others [9]. More recent studies have been performed to identify alternative P_4_ receptors, leading to the identification of two additional protein families that are able to transduce the P_4_ effects in the neuroimmune system and other tissues [10,11]: (1) Class II progestin and adipoQ receptor family members (PAQR) (such as mPRα is also known as PAQR7)); (2) B5-like heme/steroid-binding protein family that groups proteins like the PGRMC1,PGRMC2, and others [10].

PGRMC1 expression is enhanced in patients suffering from different types of tumors, resulting in poor prognosis [12,13]. In line with this observation, PGRMC1 has been linked with lipid metabolism, which would lead to enhanced breast cancer progression [14,15]. It is worth mentioning that the role of PGRMC1 is mainly evidenced in the luminal type A breast cancer cell lines (MCF7 and T47-D); meanwhile, MDA-MB-231 cell proliferation remains unaltered, even when PGRMC1 expression is artificially altered [14].

The intracellular signaling pathways activated or regulated by PGRMC1 remain barely investigated. SRC family kinase, Protein Kinase A (PKA), PKC, PI3K, and ERK-1/2 are suggested as downstream PGRMC1-dependent signaling pathways [16].

Finally, changes in the cytosolic free Ca^2+^ concentration ([Ca^2+^]_c_) have been reported to be relevant for cell proliferation [17]. In this sense, although P_4_ evokes changes in the intracellular calcium homeostasis, scientists disagree in the possible cellular Ca^2+^ sources. Thus, in retinal Muller glia cells, a non-permeable P_4_ analog evokes changes in the intracellular Ca^2+^ concentration, and further, authors reported a substantial calcium entry from the extracellular medium downstream of progesterone membrane receptors activation, but they did not show intracellular store calcium release [18]. Contrarily, human spermatozoa exhibit Ca^2+^ release from intracellular stores in response to P_4_ [19]. In neurons, P_4_ administration enhances Ca^2+^ release by favoring inositol trisphosphate receptor (IP_3_R) permeability via enhanced phosphorylation by the serine/threonine-protein kinases (AKT) [20]. Similarly, in oral squamous cancer cells (PE/CA-PJ15 cells), it has been described that P_4_ (5 and 10 μM) evokes both Ca^2+^ release and Ca^2+^ entry, which is inhibited by adding the PGRMC1 antagonist AG205 [21]. Contrarily, in the breast cancer luminal A type, T47-D, ATP evokes a transient increase in the [Ca^2+^]_c_ that is downregulated by P_4_, medroxyprogesterone acetate (MPA), and also by the progesterone non-permeable analog, progesterone-CMO-BSA. The negative effect of P_4_ on ATP-evoked calcium mobilization is non-detected in CHO cells, and experimental data has revealed the absence of mPRs in these cells [22].

Here, we aimed to extend the knowledge regarding the possible regulation of P_4_ on calcium homeostasis in breast cancer cells. We provided evidence of a novel P_4_-activated pathway in TNBC (MDA-MB-231, BT-20, and MDA-MB-468 cells). P_4_ evoked Ca^2+^ entry in MDA-MB-231 cells and MCF-7 cells by the activation of a novel calcium pathway involving the Calcium release-activated calcium channel protein 1 (Orai1), Transient receptor potential channel 1 (TRPC1), and Stromal interaction molecule 2 (STIM2); meanwhile, STIM1 silencing had no effect in this mechanism. Interestingly, activation of PGRMC1 by P_4_ attenuated this Ca^2+^ signaling pathway, which led to a decreased Ca^2+^-dependent Nuclear Factor of Activated T-Cells 1 (NFAT1) nuclear accumulation and, therefore, negatively regulated MDA-MB-231 cells proliferation.

## 2. Results

### 2.1. P_4_ Inhibits MDA-MB-231 Cell Proliferation

**Figure 1:**
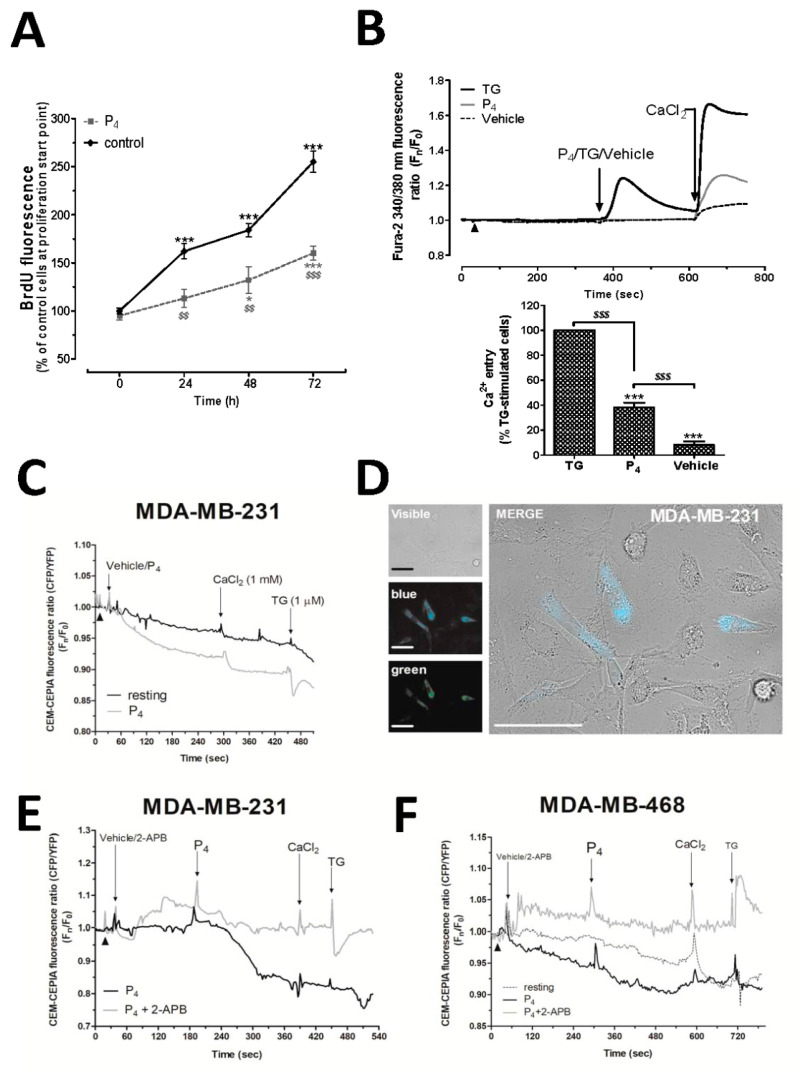
Progesterone (P_4_) altered MDA-MB-231 cell proliferation and calcium (Ca^2+^) entry. (**A**) MDA-MB-231 cells were seeded (5 × 10^5^ cells/mL) and allowed to proliferate for 72 h in the presence of the vehicle (control) or 1 μM of P_4_. At 0, 24, 48, and 72 h, cells were incubated with 5-bromo-2′-deoxyuridine (BrdU) for an additional 2 h, as described in Materials and Methods. The graph represents BrdU accumulation within the cell nucleus. *, ***: represent *p* < 0.05 and *p* < 0.001 with respect to the values of BrdU found in control cells analyzed at 0 h; *^$$^*, *^$$$^*: represent *p* < 0.01 and *p* < 0.001 with respect to the control cells at each analyzed time point. (**B**) Fura-2-loaded MDA-MB-231 cells were maintained in an extracellular Ca^2+^-free HBS medium (75 μM of ethylene glycol-bis(β-aminoethyl ether)-*N*,*N*,*N*′,*N*′-tetraacetic acid (EGTA) was added, as indicated by arrowhead), and they were subsequently stimulated either with the vehicle or P_4_ (1 μM) or thapsigargin (TG; 2 μM). Ca^2+^ release from intracellular stores upon cell stimulation was monitored for 4 min; meanwhile, Ca^2+^ entry evoked by the depletion of the intracellular Ca^2+^ stores using the different stimuli was visualized by adding CaCl_2_ (1 mM) to the extracellular medium, which was monitored for additional 2 min. Graphs are representative of 6–8 independent experiments, and the histogram represents the average of the percentage ± Standard Error of the Mean (S.E.M.), resulting in the analysis of the areas under the curves corresponding to the Ca^2+^ entry evoked by the agonist and compared to the TG-evoked Ca^2+^-entry. The 60–80 cells from 6–8 independent experiments were analyzed in these experiments. ***: represents *p* < 0.001 with respect to the percentage of Ca^2+^ values found in MDA-MB-231 cells stimulated with TG; *^$$$^*: represents *p* < 0.001 with respect to the percentage of Ca^2+^ values found in non-stimulated cells (Vehicle). (**C**–**E**) MDA-MB-231 cells (**C**–**E**) and MDA-MB-468 cells (**F**) were transfected with the intraluminal Ca^2+^ dye, GEM-CEPIA1er, and at the day of the experiments upon confirmation of the positive Ca^2+^ dye expression by the cells (**D**), the cells were maintained in a Ca^2+^-free HBS medium (75 μM of EGTA was added, as indicated by the arrowheads), and subsequently, they were stimulated with P_4_ (1 μM) in the absence (**C**) or presence of 50 μM of 2-APB (**E**,**F**), and we alternatively monitored the changes in the fluorescence of GEM-CEPIA1er at the wavelengths of 380/510 nm (excitation: Ex/emission: Em; green) and 380/462 (Ex/Em; blue). Finally, we added TG (1 μM) at the end of each experiment, which was used as an internal experimental control. Graphs and images in (**C**–**F)** are representative of 3–4 independent cell transfections with GEM-CEPIA1er. The bar in the image (**D**) represents 50 μm.

MDA-MB-231 cells lack the classical nPR [6], while these cells express a less characterized and alternatively spliced nPR, which may also bind to P_4_ [23]. Additionally, the effect of P_4_ in cell proliferation has been shown to be different according to the cell types investigated [6,8,24]. As previously reported [6], and as depicted in Figure 1A, the treatment of MDA-MB-231 cells with 1 μM of P_4_ resulted in a significant reduction of bromodeoxiuridin (BrdU) fluorescence at all analyzed times (24–72 h), as compared to cells treated with the vehicle. Therefore, these data revealed a negative role of P_4_ in the proliferation of the MDA-MB-231 cells (*n* = 6).

Considering the relevant role of the Ca^2+^- homeostasis on cell proliferation, even in MDA-MB-231 cells, as previously demonstrated by our research group [17,25,26], and the controversy regarding the role of P_4_ in calcium homeostasis aforementioned, we analyzed the effect of P_4_ on the Ca^2+^ homeostasis in MDA-MB-231 cells [18]. MDA-MB-231 cells suspended in a Ca^2+^-free medium exhibited a small Ca^2+^ entry when it was added back to the extracellular medium (1 mM CaCl_2_; Figure 1B, dotted line). Incubation of MDA-MB-231 cells with P_4_ evoked Ca^2+^ entry (Figure 1B, grey line), which was significantly smaller than the one evoked by thapsigargin in these cells (2 μM; TG, black line, Figure 1B, *p* < 0.001). TG blocks the sarcoendoplamic reticulum Ca^2+^-ATPase (SERCA), which is a classic maneuver to activate store-operated Ca^2+^ entry (SOCE) (Figure 1B, *p* < 0.001, *n* = 6) [27,28]. Interestingly, we were unable to visualize a significant P_4_-induced Ca^2+^ release from the internal stores, contrary to the results observed using TG, and therefore, it may explain the smaller activation of the Ca^2+^ entry recorded under these experimental conditions if compared to the one evoked using TG.

In order to investigate the mechanism underlying the P_4_-dependent Ca^2+^ entry activation, we analyzed possible changes in the intraluminal Ca^2+^ concentration induced by P_4_ using the endoplasmic reticulum Ca^2+^ dye, GEM-CEPIA1er, in the triple-negative breast cancer cell lines—MDA-MB-231 and MDA-MB-468 cells. As depicted in Figure 1C,D, MDA-MB-231 cells suspended in a Ca^2+^-free HBS medium presented changes in the decay of the blue/green fluorescence ratio of GEM-CEPIA1er after cell stimulation with P_4_ (1 μM) with respect to the non-stimulated resting cells (compare black and grey lines in Figure 1C). As shown in Figure 1F, similar P_4_-evoked Ca^2+^ release occurred in MDA-MB-468 cells (compare black-dotted and black-solid lines in Figure 1F). Internal control experiments consisted of the addition of TG (1 μM) at the end of each experiment, which enhanced the release of Ca^2+^ from the endoplasmic reticulum, as detected by the drastic change in the GEM-CEPIA1er fluorescence ratio. Following this, in an attempt to avoid the Ca^2+^ release evoked by P_4_, we used a relative specific antagonist of the inositol trisphosphate receptor (IP_3_R), 2-Aminoethoxydiphenyl borate (2-APB), in MDA-MB-231 cells (Figure 1E) and MDA-MB-468 cells (Figure 1F) [29]. As shown in Figure 1E,F, treatment of the triple-negative breast cancer cells with 50 μM of 2-APB for 5 min was able to almost completely block the release of Ca^2+^ evoked by P_4_. Therefore, these results allowed us to conclude that although not being reported using fura-2, P_4_ evokes enough intraluminal Ca^2+^ changes to promote the extracellular Ca^2+^ entry, and, further, the Ca^2+^ release evoked by P_4_ occurs through the IP_3_R.

### 2.2. Activation of PGRMC1 Downregulates P_4_-Dependent Ca^2+^ Mobilization in MDA-MB-231 Cells

**Figure 2:**
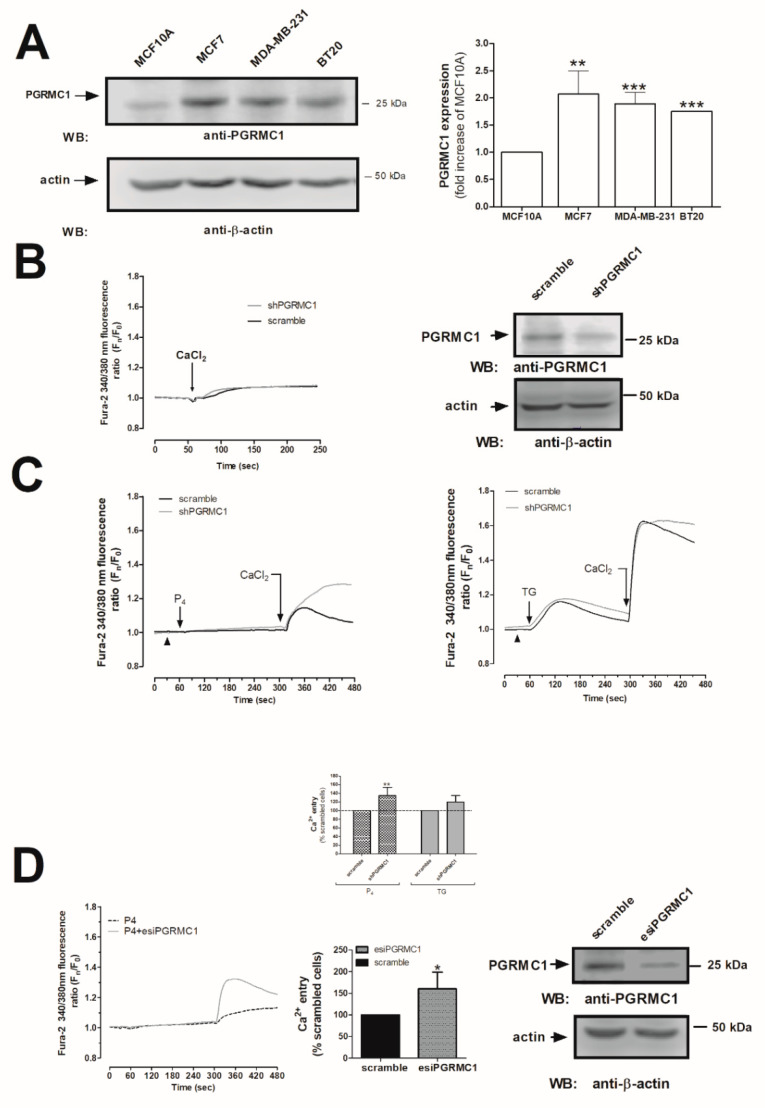
Progesterone receptor membrane component 1 (PGRMC1) downregulated the progesterone (P_4_)-evoked Ca^2+^ entry. (**A**) Resting MCF10A, MCF7, MDA-MB-231, and BT20 cells were suspended at the same concentration, and they were subsequently lysed with NP40 buffer. The resulting protein samples were denaturalized by mixing with an equal volume of Laemmli’s buffer (5% sodium dodecyl sulphate, SDS). Subsequent Western blotting was performed by using the anti-PGRMC1 antibody, as described in Materials and Methods. The histogram represents the average of the fold increase ± S.E.M. of the amount of PGRMC1 found in MCF10A after they were previously normalized by considering the actin content in each gel lane. **, ***: represent *p* < 0.01 and *p* < 0.001 as compared to the PGRMC1 values found in MCF10A cells. (**B**,**C**) MDA-MB-231 cells were transfected for 48 h with 2 μg/mL of the small hairpin siRNA against PGRMC1 (shPGRMC1) plasmid (**B**) or esiPGRMC1 (**D**), and after validation of the efficiency of the silencing procedure by Western blotting, cells were incubated with 2 μM of fura-2/AM for 30 min at 37 °C. (**B**) In order to demonstrate that the PGRMC1 silencing did not affect Ca^2+^-mobilization per se in MDA-MB-231 cells, 1 mM of CaCl_2_ was extracellularly added to cells under resting conditions. (**C**) MDA-MB-231 cells transfected with the empty vector (scramble) or with shPGRMC1 and were maintained in a Ca^2+^-free HBS medium (75 μM of ethylene glycol-bis(β-aminoethyl ether)-*N*,*N*,*N*′,*N*′-tetraacetic acid (EGTA) was added, arrowheads), and finally, they were stimulated either with 1 μM of P_4_ or 2 μM of TG. Additionally, P_4_ or thapsigargin (TG)-evoked Ca^2+^ entry was visualized by adding CaCl_2_ (1 mM) to the extracellular medium, which was monitored for an additional 2 min. (**D**) MDA-MB-231 cells were transfected with esiPGRMC1 for 48 h, and subsequently, P_4_-evoked Ca^2+^ mobilization was analyzed by using similar experimental conditions to those used in (**C**). Graphs are representative of 6–8 independent experiments, and the histogram represents the average of the percentage ± Standard Error of the Mean (S.E.M.), resulting in the analysis of the areas under the curves corresponding to the Ca^2+^ entry evoked by P_4_ or TG. The 60–80 cells from 6–8 independent transfections were considered during the analysis. *: represents *p* < 0.05; **: represents *p* < 0.01 with respect to the Ca^2+^ entry values found in scrambled MDA-MB-231 cells.

**Figure 3:**
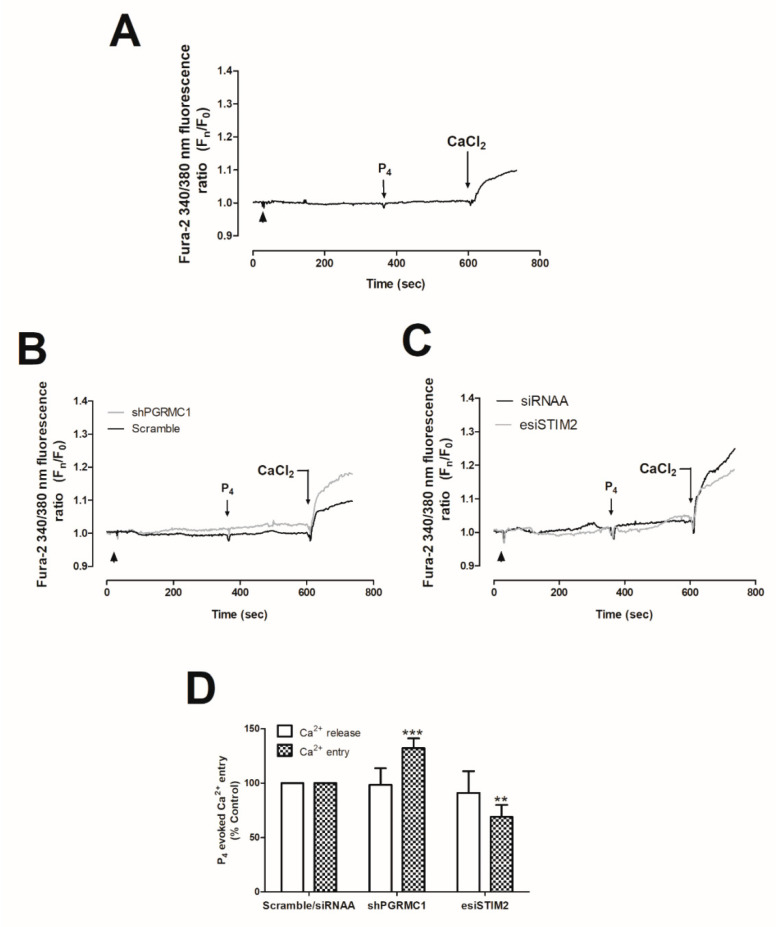
Progesterone (P_4_) effects on Ca^2+^ entry in MCF7 mock cells or in cells transfected with small hairpin siRNA against progesterone receptor membrane component 1 (shPGRMC1) and endoribonuclease-prepared siRNAs against the stromal interaction molecular 2 (esiSTIM2). MCF7 cells were kept untreated (**A**) or were transfected for 48 h with 2 μg/mL of either shPGRMC1 plasmid (**B**), esiSTIM2 (**C**), or their respective controls, and upon confirming the efficiency of the genetic modification, cells were incubated with 2 μM of fura-2/AM for 30 min at 37 °C. Cells were finally maintained in a Ca^2+^-free HBS medium (75 μM of ethylene glycol-bis(β-aminoethyl ether)-*N*,*N*,*N*′,*N*′-tetraacetic acid (EGTA) was added, arrowheads) and were stimulated with 1 μM of P_4_ for 4 min. P_4_-evoked Ca^2+^ entry was visualized by adding CaCl_2_ (1 mM) to the extracellular medium, which was further monitored for an additional 2 min. Graphs are representative of 4–6 independent experiments. (**D**) The histogram represents the average of the percentage ± Standard Error of the Mean (S.E.M.), resulting in the analysis of the areas under the curves corresponding to the Ca^2+^-entry obtained using shPGRMC1 and esiSTIM2 with respect to scrambled cells. The 20–40 cells from 4–6 independent transfections were considered during the analysis. **, ***: represent *p* < 0.01 and *p* < 0.001 with respect to the Ca^2+^ mobilization values found in scrambled MCF7 cells stimulated with P_4_.

As shown in Figure 2A, MDA-MB-231 cells presented greater PGRMC1 expression values as compared to the non-tumoral cell line, MCF10A (1.9 ± 0.2-fold increase as compared to MCF10A, *p* < 0.001, respectively; *n* = 6). Similarly, enhanced PGRMC1 expression was detected in other breast cancer cell types—the luminal A type MCF7 and triple-negative BT-20 (Figure 2A, *p* < 0.01 and *p* < 0.001, respectively). In addition, considering the discrepancies mentioned above regarding the structural identity of the P_4_-receptor in the MDA-MB-231 cells, we silenced the expression of the PGRMC1 receptor by using the small hairpin RNA against PGRMC1 (shPGRMC1) and an endoribonuclease-prepared siRNAs against PGRMC1 (esiPGRMC1). MDA-MB-231 cell transfection with either shPGRMC1 or esiPGRMC1 efficiently reduced PGRMC1 expression in MDA-MB-231 cells after 48 h of transfection (See WB image in Figure 2B,D). Interestingly, PGRMC1 silencing did not alter Ca^2+^ mobilization in resting cells (Figure 2B, Ca^2+^ traces), but an increase in the P_4_-induced Ca^2+^ entry by a 35 ± 19% (using ShPGRMC1; Figure 2C) and 60 ± 38% (using esiPGRMC1; Figure 2D) was observed as compared to the non-genetically modified MDA-MB-231 cells (see cells transfected with scramble plasmids vs. shPGRMC1 and esiPGRMC1, respectively, and the histogram at the bottom of Figure 2C,D; *p* < 0.01 and *p* < 0.05, *n* = 6). On the contrary, we did not observe significant changes in the TG-evoked SOCE in MDA-MB-231 cells transfected with shPGRMC1 under these experimental conditions (Figure 2C, right-hand side graph, 20 ± 15%; *p* > 0.05, *n* = 6). According to our results, stimulation of Ca^2+^ entry by P_4_ was downregulated by PGRMC1 in MDA-MB-231 cells, and the latter results were also reproduced in MCF7 cells (See Figure 3A,B,D). These results agreed with the downregulation of (Ca^2+^)_c_ concentration by P_4_, as previously described in spontaneously immortalized rat granulosa cells and breast cancer luminal A type, T47-D, but authors did not deeply explore this mechanism [22,30].

STIM1 is a very relevant component of the Ca^2+^ entry in non-electrical excitable cells, including breast cancer cells [31]. In order to dig into the mechanism underlying the P_4_-evoked changes in Ca^2+^ entry, we knocked-down STIM1 by using esiRNA [28,32,33,34,35]. esiRNA consists of a commercial combination of siRNAs directed against different sequences of the targeted protein that has been generated by endonuclease activity, and thus, they would present greater efficiency and specificity than siRNAs, which are generated toward a unique sequence of the targeted protein [36].

**Figure 4:**
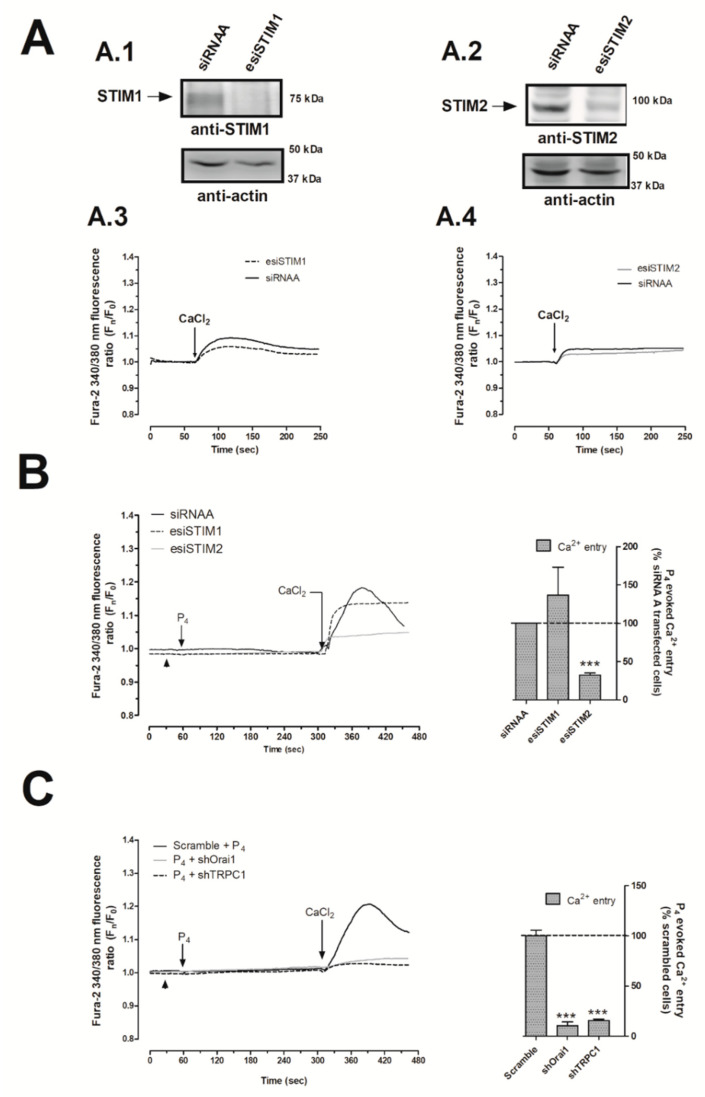
Progesterone (P_4_) activated the stromal interaction molecule 2 (STIM2) that drives calcium (Ca^2+^) entry through Calcium release-activated calcium channel protein 1 (Orai1) and transient receptor potential channel 1 (TRPC1). MDA-MB-231 cells were transfected with 2 μg/mL of either endoribonuclease-generated siRNA against STIM1 (esiSTIM1) (**A.1**,**A.3** (dotted trace) and **B**) or esiSTIM2 (**A.2**,**A.4** (grey trace) and **B**) or with a non-specific siRNA (**A.1**–**A.4** (black trace) and **B**) for 48 h. Alternatively, we used 2 μg/mL of small hairpin RNA against Orai1 (shOrai1) (**C**, grey solid line) or shTRPC1 (**c**; black dotted line) or empty vector (**C**, scrambled cells, black trace) for 48 h. Upon confirmation of the efficiency of the protein silencing protocols by Western blotting using the specific antibodies as indicated (**A.1**,**A.2**), Ca^2+^ experiments were performed according to the procedures described in Materials and Methods. Briefly, fura-2-loaded MDA-MB-231 cells were maintained in a Ca^2+^-rich medium (containing 1 mM of CaCl_2_, **A.3**,**A.4**) and were kept untreated; while other cells were maintained in a Ca^2+^-free HBS medium (75 μM of ethylene glycol-bis(β-aminoethyl ether)-*N*,*N*,*N*′,*N*′-tetraacetic acid (EGTA) was added, arrowheads (**B**,**C**)) and were stimulated with 1 μM of P_4_ for 4 min (**B**,**C**). P_4_-evoked Ca^2+^ entry was visualized by adding CaCl_2_ (1 mM) to the extracellular medium, which was monitored for an additional 2 min. Graphs are representative of 6–8 independent transfections, and the histograms represent the average of the percentage ± Standar error of the Mean (S.E.M.), resulting in the analysis of the areas under the curves corresponding to the Ca^2+^ entry evoked by P_4_ in around 60–80 cells from 6–8 independent transfections. ***: represents *p* < 0.001 with respect to the Ca^2+^ entry values found in scrambled MDA-MB-231 cells.

As depicted in Figure 4A.1, transfection of MDA-MB-231 cells with esiSTIM1 completely silenced STIM1 expression, which did not significantly alter the Ca^2+^ homeostasis of MDA-MB-231 cells maintained under normal conditions (Figure 4A.3). In our hands, esiSTIM1 did not quantitatively modify the Ca^2+^ entry induced by the administration of P_4_ (Figure 4B, black-dotted trace; *p* > 0.05, *n* = 6). Next, we explored the possible role of STIM2, a less characterized isoform of STIM1 [37,38], which is able to detect smaller changes in the endoplasmic reticulum’s Ca^2+^ content in response to low physiological agonist concentration, and therefore, it may detect the small P_4_-evoked intraluminal Ca^2+^ changes, previously shown in Figure 1 [39,40,41]. The transfection of MDA-MB-231 cells with the esiSTIM2 significantly reduced the expression of the targeted protein (Figure 4A.2), and as evidenced in Figure 4B, STIM2 silencing attenuated P_4_-evoked Ca^2+^ entry by 67.8 ± 3.0% (Figure 4B, grey trace; *p* < 0.001, *n* = 6) but did not significantly modify the Ca^2+^ homeostasis in non-stimulated cells (Figure 4A.4). Similarly, the downregulation of P_4_-evoked Ca^2+^ entry was observed in MCF7 lacking STIM2 (Figure 3C,D).

**Figure 5:**
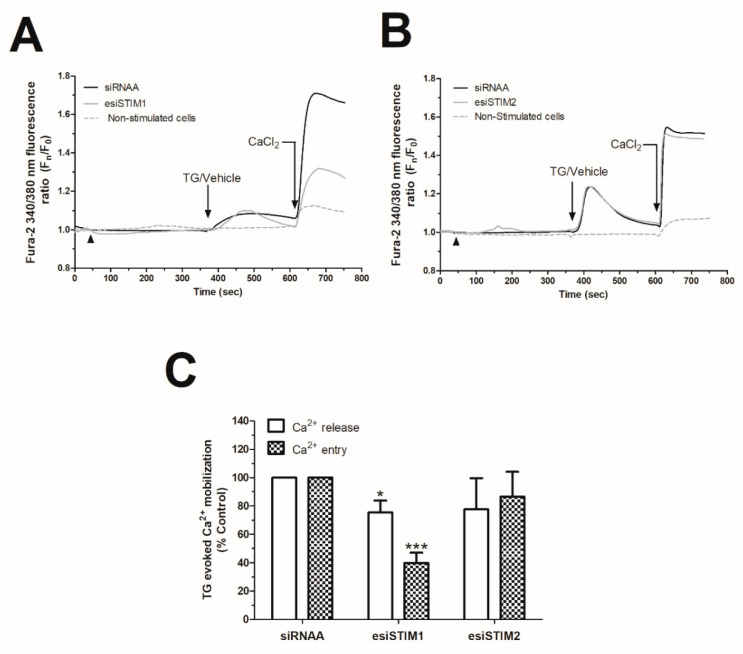
Effect of stromal interaction molecule 1 (STIM1) and STIM2 silencing on thapsigargin (TG)-evoked store-operated Ca^2+^ entry (SOCE) in MDA-MB-231 cells. MDA-MB-231 cells were transfected with either endonuclease-generated siRNA against STIM1 (esiSTIM1) (**A**) and esiSTIM2 (**B**) or siRNA (**A**,**B**). Ca^2+^ experiments under single-cell imaging configuration were performed by loading cells with fura-2/AM. Fura-2-loaded cells were maintained in a Ca^2+^-free HBS medium (75 μM of ethylene glycol-bis(β-aminoethyl ether)-*N*,*N*,*N*′,*N*′-tetraacetic acid (EGTA) was added, arrowheads) and were stimulated with 2 μM of TG or treated with the vehicle for 4 min, while we monitored the TG-evoked Ca^2+^ release. Subsequently, TG-evoked Ca^2+^ entry (SOCE) and constitutive Ca^2+^ entry were visualized by adding CaCl_2_ (1 mM) to the extracellular medium and were monitored for an additional 2 min. Graphs are representative of 6–8 independent transfections. (**C**) The histogram represents the average of the percentage ± Standar Erro of the Mean S.E.M., resulting in the analysis of the areas under the curves evoked by TG in around 60–80 cells from 6–8 independent transfections. *, ***: represent *p* < 0.05 and *p* < 0.001 with respect to the Ca^2+^ mobilization values found in scrambled MDA-MB-231 cells.

Following this, we explored the plasma-membrane Ca^2+^ channels activated by P_4_ in MDA-MB-231 cells. Thus, the transfection of MDA-MB-231 cells with shRNAs against Orai1 and TRPC1 channels resulted in a significant reduction (89.5 ± 3.8% and 84.3 ± 1.2%) in P_4_-evoked Ca^2+^ entry as compared to the cells transfected with scrambled plasmids (Figure 4C; *p* < 0.001; *n* = 6). Therefore, our findings indicate that the P_4_-evoked Ca^2+^ entry course through a novel pathway where STIM2, Orai1, and TRPC1 are involved. Interestingly, STIM2 silencing did not alter the activation of SOCE in MDA-MB-231 cells, which is mainly conducted by STIM1 and Orai1, as depicted in Figure 5A–C, and it has been previously reported by others [31].

**Figure 6:**
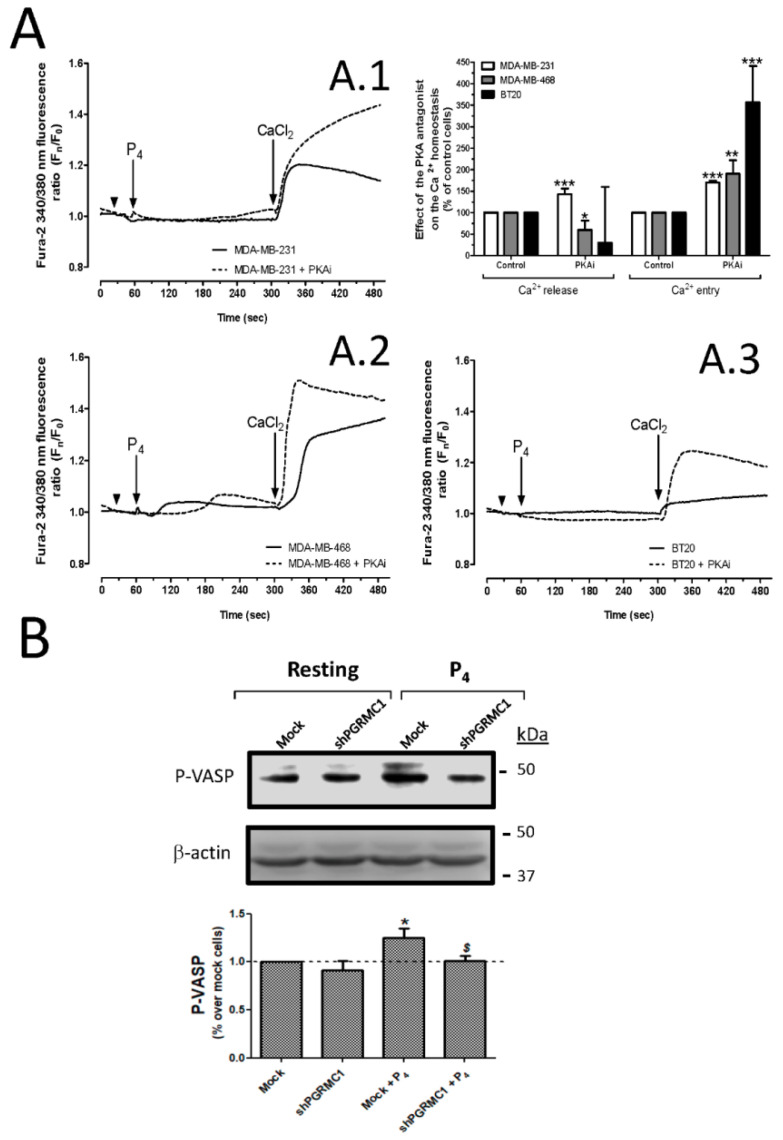
Protein kinase A (PKA) activation by progesterone (P_4_) impaired calcium (Ca^2+^) entry in triple-negative breast cancer cells. MDA-MB-231 cells (**A.1**), MDA-MB-468 (**A.2**), and BT20 cells (**A.3**) were loaded with fura-2/AM. Single-cell imaging experiments were done using fura-2 loaded triple-negative breast cancer cells that were previously incubated for 30 min with the vehicle (black solid traces) or with 3 μM of the PKA antagonist, KT5720 (PKAi, black dotted traces). Following this, the triple-negative breast cancer cells were stimulated for 4 min with P_4_ (1 μM) in a Ca^2+^-free HBS medium (75 μM of ethylene glycol-bis(β-aminoethyl ether)-N,N,N′,N′-tetraacetic acid (EGTA) was added, as indicated by arrowheads), and subsequently, P_4_-evoked Ca^2+^ entry was visualized by adding 1 mM of CaCl_2_ to the extracellular medium. Areas under the curves evoked by P_4_ and CaCl_2_ administration to the cells were determined, and they were used for comparison between both experimental conditions. The histogram represents the average of the percentage ± Standard error of the Mean (S.E.M.), resulting in the analysis of the areas under the curves corresponding to the P_4_-evoked Ca^2+^ release and Ca^2+^ entry evoked in the absence or presence of the PKA antagonist. The 20-30 cells from 6 independent experiments were considered during the analysis. *, **, ***: represent *p* < 0.05, *p* < 0.01, and *p* < 0.001 with respect to the control PKAi untreated cells, respectively. (**B**) The activation of PKA was analyzed by determining the phosphorylation rates of vasodilator stimulated phosphoprotein (VASP; at the Ser239) in MDA-MB-231 cells lacking or expressing the progesterone receptor membrane component 1 (PGRMC1), which were subsequently stimulated for 1 min with the vehicle or 1 μM of P_4_. The image is representative of 3 independent transfections, and the bar graph represents the increase in the phosphorylation rate of VASP with respect to the control mock cells under resting conditions. *: represents *p* < 0.05; meanwhile, *^$^*: represents *p* < 0.05 with respect to the cell stimulated with P_4_ but non-transfected with the small hairpin siRNA against PGMRC1 (shPGRMC1).

Structural analysis of the Orai1 structure revealed that Orai1 is phosphorylated at the serine-34 residue by the protein kinase A (PKA), which modulates the channel function [42]. Furthermore, it has been described that activation of PGRMC1 leads to the activation of certain protein kinases like PKA, PI3K, and SFK [16]. Therefore, we explored whether the inhibition of P_4_-evoked Ca^2+^ entry might occur through the activation of the PGRMC1/cAMP/PKA signaling pathways [43,44]. As depicted in Figure 6, MDA-MB-231 (6A.1), MDA-MB-468 (6A.2), and BT20 (6A.3) cells were preincubated with 3 μM of the specific PKA antagonist, KT5720 (PKAi), which resulted in a significant increase in the P_4_-evoked Ca^2+^ entry, as summarized in the bar graph in Figure 6A. Additionally, to further demonstrate the activation of PKA in MDA-MB-231 cells in response to P_4_ stimulation, we analyzed the changes in the phosphorylation at Ser239 of vasodilator stimulated phosphoprotein (VASP) that is a substrate of PKA and has commonly been used for monitoring the PKA activation. As shown in Figure 6B, P_4_ evoked a 1.3 ± 0.06-fold increase in P-VASP as compared to mock resting cells (*p* < 0.05; *n* = 3); meanwhile, PGRMC1 silencing reversed P-VASP to similar values than those found in mock resting cells (*p* < 0.05; *n* = 3). These data reproduced the observation noted with the shPGRMC1 (*n* = 6), and therefore, led us to conclude that PGRMC1 impairs P_4_-induced Ca^2+^ entry by activating PKA and, subsequently, by inducing Orai1 phosphorylation, as it has been previously demonstrated by others [42].

### 2.3. PGRMC1 Silencing Reduces the Negative Effect of P_4_ in MDA-MB-231 Cell Proliferation

**Figure 7:**
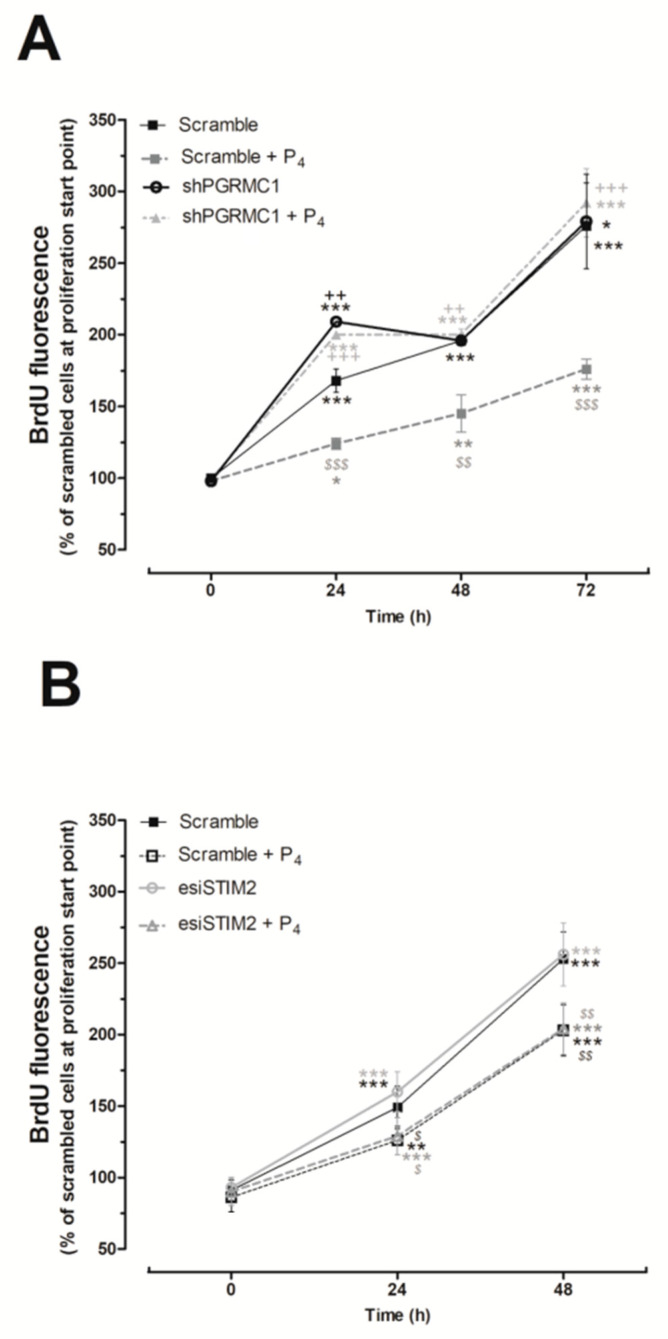
Effect of silencing the progesterone receptor membrane component 1 (PGRMC1) and the stromal interaction molecule 2 (STIM2) in the proliferation of MDA-MB-231 cells. MDA-MB-231 cells were transfected for 48 h with either small hairpin siRNA against PGRMC1 (shPGRMC1) (**A**) or the respective empty vector (**A**; scramble), and alternatively, cells were transfected with either endoribonuclease-generated siRNA against STIM2 (esiSTIM2) (**B**) or siRNA for 48 h (**B**; scramble). Upon confirming the efficiency of the silencing protocol by Western blotting, the cells were seeded and allowed to proliferate in the absence or presence of progesterone (P_4_; 1 μM)_._ The proliferation rate was determined by analyzing the bromodeoxyuridine (BrdU) uptake by the cells for 2 h at 0, 24, 48, and 72 h from the beginning of the proliferation experiments. Graphs represent the average of the percentage ± Standard of the Mean (S.E.M.) of the BrdU fluorescence detected in the scrambled cells at the beginning of the cell culture (time 0 h). *, **, ***: represent *p* < 0.05, *p* < 0.01, and *p* < 0.001 with respect to the scrambled cells at time 0 h; meanwhile, *^$^*,*^$$^*, *^$$$^*: represent *p* < 0.05, *p* < 0.01, and *p* < 0.001 with respect to the BrdU values found in scrambled cells grown in the absence of P_4_ at the different analyzed time points, and ^++^,^+++^: represent *p* < 0.01 and *p* < 0.001 with respect to the values found in cells expressing the targeted protein.

A positive effect of P_4_ in proliferation has been reported in cells expressing the classical nPR, such as MCF7 or glioblastoma cells [45,46,47]; meanwhile, the negative effect of P_4_ on cell proliferation was observed in cells lacking the nPR like the human BxPC3 pancreatic adenocarcinoma cells [48]. In TNBC cells, we explored the role of PGRMC1 on the P_4-_mediated inhibition of cell proliferation, previously described in Figure 1. As shown in Figure 7A, the silencing of PGRMC1 reversed the inhibitory effect of P_4_ on MDA-MB-231 cell proliferation, thus suggesting that this negative effect is mediated by the P_4_ binding and activation of PGRMC1. Furthermore, our results indicated that the silencing of STIM2 did not alter the proliferation pattern found in MDA-MB-231 cultured in the presence of P_4_ for 24 h (See Figure 7B); then, STIM2 would not be involved in the negative role of P_4_ on MDA-MB-231 cells proliferation.

### 2.4. PGRMC1 Is Involved in the P_4_-Evoked Cell Death in Breast Cancer Cells

**Figure 8:**
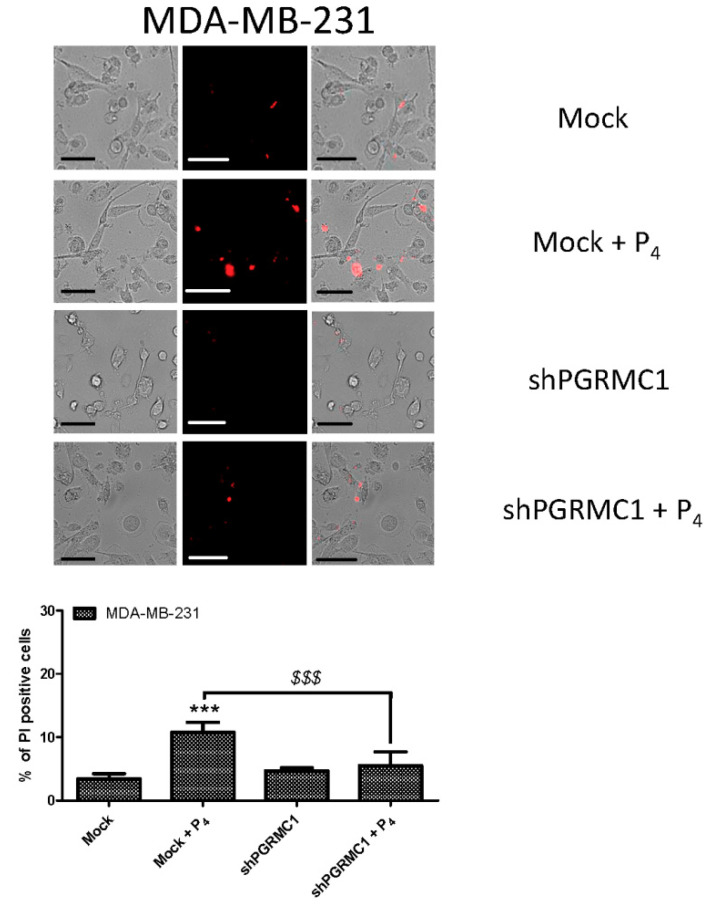
Protesterone (P_4_) induced cell death of the MDA-MB-231 cells. MDA-MB-231 cells were transfected with the scrambled (Mock) or the small hairpin siRNA against the progesterone receptor membrane component 1 (shPGRMC1) plasmids for 48 h. After confirmation of the efficiency of the silencing protocol by Western blotting, cells were cultured for an additional 24 h in the absence or presence of P_4_ (1 μM). Following this, cells were incubated on the day of the experiment with 4 μM of propidium iodide (PI) for 45 min at 37 °C inside the incubator. Cell images were acquired at 555/624 nm of wavelength (excitation/emission, respectively), by using a 40× objective mounted in a fluorescence microscope. The percentage of positive PI loaded cells with respect to the total counted cells in the fields was estimated, considering the mean ± Standard error of the mean (S.E.M.) of 3–4 independent experiments, and was represented in the bar chart. ***: represents *p* < 0.001 with respect to the mock resting cells; meanwhile, *^$$$^*: represents *p* < 0.001 with respect to the cells expressing PGRMC1 and treated with P_4_. The bar in the images represents 50 μm.

In order to confirm that the negative effect of P_4_ on breast cancer cells course by activation of PGRMC1, we analyzed the number of dead cells by using the PI uptake protocol in MDA-MB-231 cells that were previously transfected with the scramble or shPGRMC1 plasmids. As shown in Figure 8, MDA-MB-231 cells exhibited a significant increase in the percentage of positive PI cells after incubation with P_4_ (1 μM) for 24 h. Interestingly, MDA-MB-231 cells transfected with shPGRMC1 presented a reduction in the percentage of dead cells after the incubation with P_4_, as compared to the scrambled control cells (Mock cells, *p* < 0.001; *n* = 3–4). These results might explain the reduction in the cell count observed at similar times that are shown in Figure 1 and Figure 4.

### 2.5. PGRMC1 Activation by P_4_ Impairs Nuclear Factor of Activated T-Cells 1 (NFAT1) Nuclear Translocation

Finally, we analyzed the effect of P_4_ on the activation of NFAT1, an important calcium-dependent and pro-proliferative transcription factor that acts as checkpoints in the cell cycle [49,50,51,52,53].

**Figure 9:**
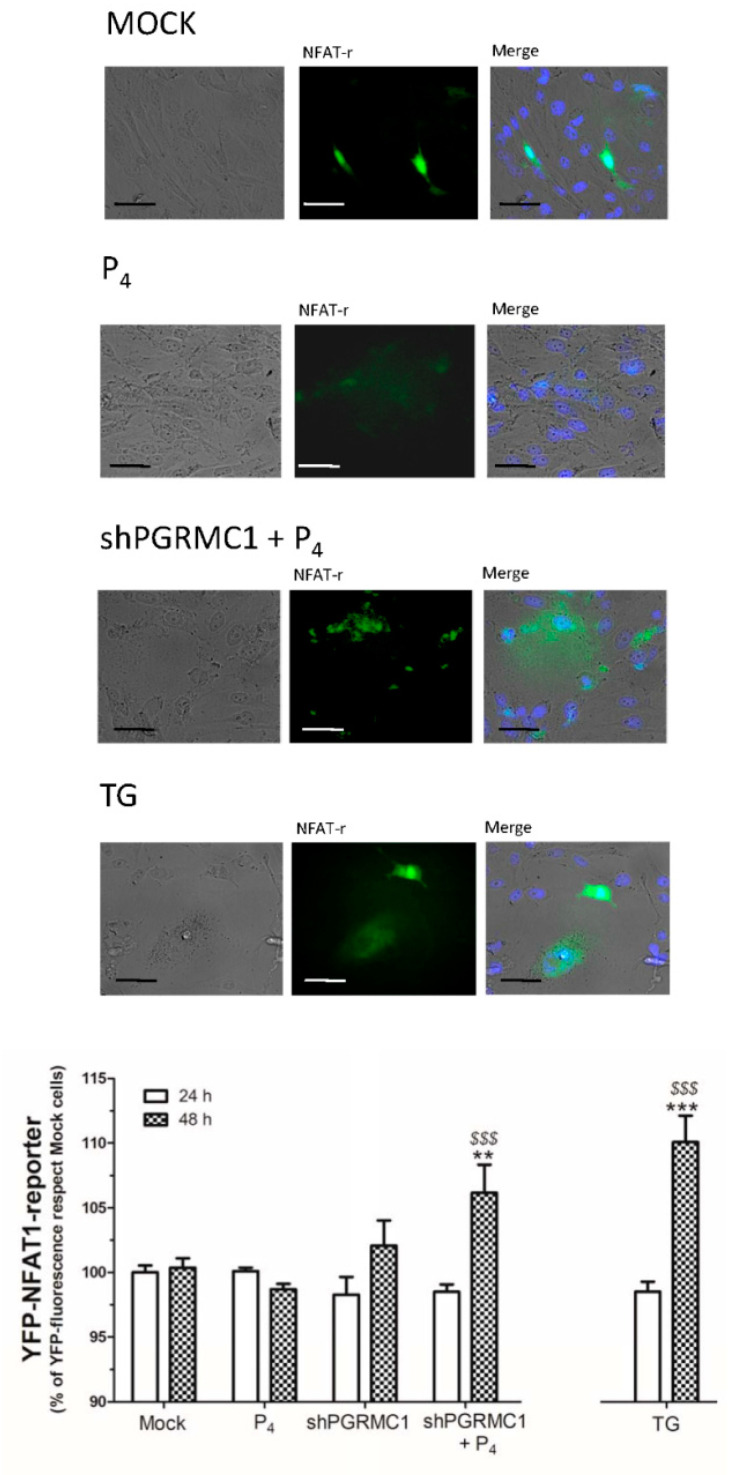
Progesterone receptor membrane component 1 (PGRMC1) silencing enhanced nuclear factor of activated T-cells 1 (NFAT1) translocation to the cell nuclei in MDA-MB-231 cells. MDA-MB-231 cells were transfected for 48 h with yellow fluorescent protein (YFP)-NFAT1-reporter plasmid alone or in combination with the small hairpin siRNA against PGRMC1 (shPGRMC1). Fluorescence of NFAT1-reporter (NFAT-r) was analyzed using cell images of the cultures kept under regular condition or after being incubated with 1 μM of progesterone (P_4_) for 24 and 48 h. Images are representative of 3 independent transfections after 48 h of P_4_ administration to the respective cell batches. The histogram represents the percentage ± Standard Error of the Mean (S.E.M.) of the NFAT1-reporter fluorescence found in 4–8 images obtained from 3 independent transfections and after normalizing using the fluorescence found in scrambled and P_4_-untreated cells (mock). Internal experimental control consisted of the quantification of NFAT1 translocation due to store-operated Calcium entry (SOCE) evoked by thapsigargin (TG) administration. **, ***: represent *p* < 0.01 and 0.001 with respect to the scrambled and P_4_-untreated cells; meanwhile, *^$$$^*: represents *p* < 0.001 with respect to the scrambled cells and cells treated with P_4_, respectively. The bar represents 50 μm.

As depicted in Figure 9, NFAT1-reporter fluorescence was significantly enhanced in cells where PGRMC1 was silenced and, subsequently, stimulated for 48 h with 1 μM of P_4_, perhaps, enhanced NFAT1 reporter visualization results of the biggest Ca^2+^ entry previously shown in these cells lacking PGRMC1 under similar experimental conditions (Figure 2), and it has been also reported by others.

## 3. Discussion

The role of P_4_ on cell proliferation has been widely debated due to the presence of different receptors that may be targeted by this hormone in the different tissues [8,9,35,49,54]. In our hands, stimulation of MDA-MB-231 cells with P_4_ (1 μM) resulted in a PGRMC1-dependent reduction of cell proliferation, as demonstrated by the observation that PGRMC1 silencing impairs the inhibitory effect of P_4_ in cell proliferation. Furthermore, P_4_-evoked Ca^2+^ entry was strongly dependent on the activation of STIM2, Orai1, and TRPC1. Furthermore, P_4_-evoked Ca^2+^ entry was bigger in cells lacking PGRMC1 and in those cells pre-incubated with the PKA antagonist KT5720, thus, suggesting that the PGRMC1 plays a negative role in the P_4_-induced Ca^2+^ signaling. PGRMC1 silencing reversed the P_4_ negative effect on the Ca^2+^ entry reported in the MDA-MB-231 cells, which would lead to an enhanced NFAT1 translocation to the nucleus, as evidenced by using the NFAT1-reporter.

MDA-MB-231 cells express at least three different types of P_4_ receptors: PAQR family, PGRMC family, and sigma-2R. The latter was initially considered to be the PGRMC1; in fact, current evidence supports this idea [55,56]. Nonetheless, sigma-2R has been recently claimed to be different from PGRMC1 based on genetic and biochemical evidence [57,58,59,60]. In MDA-MB-231 cells, we demonstrated that inhibition of sigma-2R with NO1 reported a different effect on TG-evoked SOCE to the one presented here by silencing PGRMC1 [26]. The different physiological roles of both proteins would confirm the previous observations done by other groups [55]; however, we could not discard that both proteins would be part of a macromolecular complex targeted by P_4_. On the other hand, regarding the possible relationship between the members of the PAQR and PGRMC1 families, controversy still remains. In this sense, it has been reported that mPRα expressed in seatrout oocyte membranes would be activated by P_4_, which would subsequently downregulate cAMP production by impairing the activation of the adenylate cyclase [54,61], but according to the results obtained with the PKA antagonist and further demonstrated with the analysis of P-VASP, we believed that the latter did not take place in our cell models. Additionally, these authors reported that PGRMC1 might be involved in this pathway since PGRMC1 supports the access of mPRα to the plasma membrane [11,60]. Contrarily, artificial human mPRα expressed in HEK293 cells and MDA-MB-231 cells has failed to evoke Ca^2+^ mobilization in response to P_4_, and it is not able to interact with G proteins, and therefore, it is not able to activate cAMP or MAPK (ERK1/2 or p38). In fact, these authors concluded that mPRα was not expressed in the plasma membrane [30]. By silencing PGRMC1, we were able to completely abolish the negative effect of P_4_ on the proliferation of the MDA-MB-231 cells, which denotes a relevant role of this protein in the physiology of the MDA-MB-231 cells, as previously indicated by others [8,10]. Interestingly, PGRMC1 knockdown in the human colon cancer cell line, HCT116, has reported the contrary effect since authors evidenced a decrease in proliferation and metastasis to the liver in cells lacking this protein [62].

Regarding the role of P_4_ in the regulation of the intracellular Ca^2+^ homeostasis, it has been reported that the hormone reduces the expression of Ca^2+^ channels and Ca^2+^-dependent proteins involved in cell contraction in mouse embryonic stem cells (such as Ryr2, Calm2, Trpv2, and Mylk3) [63]. In line with this observation, spontaneously immortalized granulosa cells exhibit a dramatic reduction in the (Ca^2+^)_c_ in response to prolonged P_4_ administration [30]. In the breast cancer cell line MCF7, P_4_ elicits changes in the expression of S100A11, S100A10, calreticulin, VDAC1, SERCA3, and SERCA1, resulting in a barely Ca^2+^ efflux from the endoplasmic reticulum as well as impaired membrane potential in the mitochondria [64]. Here, we reported for the very first time that P_4_-evoked Ca^2+^ entry requires STIM2 activation in MDA-MB-231 and MCF7 cell lines (Figure 3 and Figure 4), which operates Ca^2+^ entry in response to small changes in the amount of Ca^2+^ accumulated in the endoplasmic reticulum [28,36]. The latter was evidenced here by using GEM-CEPIA1er, a Ca^2+^ dye that was specifically designed to monitor the intraluminal Ca^2+^ changes in the transfected cells [65]. Interestingly, the combined use of this Ca^2+^ dye and 2-APB allowed us to demonstrate that in the triple-negative breast cancer cells (MDA-MB-231 and MDA-MB-468), the release of Ca^2+^ evoked by P_4_ occurred through the IP_3_R (Figure 1). 2-APB was previously reported to be a relatively high specific IP_3_R type 1 antagonist, but it may also affect IP_3_R type 3 when used at very elevated concentrations [29]. P_4_-evoked Ca^2+^ entry was also found to be dependent on Orai1 and TRPC1 activation. Therefore, PGRMC1 silencing enhanced P_4_-evoked Ca^2+^ entry and reversed the P_4_ inhibitory role in cell proliferation. In line with this observation, the antagonist of PKA, KT5720, enhanced the P_4_-evoked Ca^2+^ entry in all the triple breast cancer cell types evaluated (Figure 6A), so it did the silencing of PGRMC1 in MDA-MB-231 cells and MCF7 cells. Additional evidence of the existence of a negative regulatory pathway downstream of PGRMC1, which depends on PKA activation results of the evaluation of the level of P-VASP, being the phosphorylation of VASP, impaired in MDA-MB-231 cells lacking PGRMC1 (See Figure 6B), might support the existence of a P_4_/PGRMC1/cAMP/PKA signaling pathway in breast cancer cells, modulating the P_4_-evoked Ca^2+^ entry via phosphorylation of Orai1 by PKA, as previously reported [43,44].

The regulation of intracellular Ca^2+^ homeostasis is crucial for the activation of several key checkpoint proteins involved in the cell cycle; hence, alteration of this Ca^2+^ fluxes might have negative effects on cell proliferation [66]. It has been demonstrated that transient Ca^2+^ currents like those conducted by voltage-operated Ca^2+^ channels activation are very effective, activating transcription factors to maintain the cells in a quiescent status [67,68]. Ca^2+^ currents through the plasma membrane, such as SOCE, have been reported to evoke the activation of the pro-proliferative factor NFAT1 and its translocation to the nucleus [52,69]. Stimulation of cardiac myocytes with P_4_ leads to NFAT1 activation that is reversed in the presence of cyclosporine, an antagonist of the Ca^2+^-dependent phosphatase calcineurin, which is the main protein responsible for NFAT1 dephosphorylation, and subsequently, it is required for NFAT1 migration to the cell nucleus [70]. Activation of this pathway has been widely described downstream of the canonical SOCE Ca^2+^ channel, Orai1 [70]. Here, in MDA-MB-231 cells, P_4_-STIM2 dependent Ca^2+^ entry conducted the activation of Orai1 and TRPC1, and it led to NFAT1 nuclear translocation, as evidenced by the increase in the fluorescence of the NFAT1 reporter. In line with this, PGRMC1 silencing favored an enhanced NFAT1 accumulation in cells treated with P_4_ (Figure 9), thus favoring cell proliferation similar to that in prostate cancer cells [71,72]. In addition, NFAT1 has been reported to be overexpressed in several breast types like pancreatic, lung, and other cancer types [73,74]. Interestingly, Ca^2+^ entry through SOCE, a mechanism that relies on Orai1/STIM1 interaction, leads to NFAT1 activation due to a calcineurin-dependent dephosphorylation mechanism, as described above. Lack of STIM2 role in proliferation has been recently described in other cancer cell lines. Contrarily, a recent paper has claimed that STIM2 is responsible for the epithelial-mesenchymal transition (EMT), while others have pointed out that it may be involved in migration and promoting metastasis [75,76,77]. So, during cancer onset, PGRMC1 might be temporally downregulated, and thus, cancer cells might avoid the negative effect of P_4_ in proliferation (shown in Figure 7 and Figure 8), and in parallel, these cells might get the benefit of the activation of NFAT1 that would allow the MDA-MB-231 cells to migrate and initiate other cancer sprouts (shown in Figure 9).

**Figure 10:**
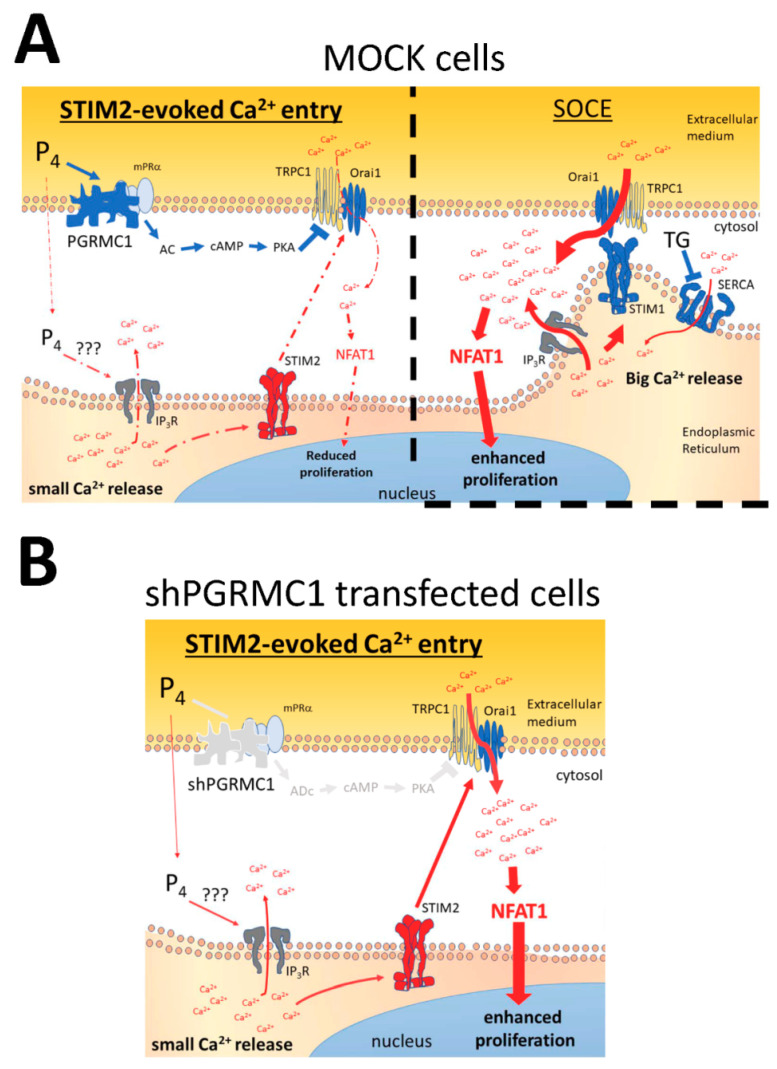
Schematic representation of the proposed mechanism of the P_4_ effects on Ca^2+^ homeostasis. (**A**) In MDA-MB-231 cells, STIM2 detects the small intraluminal Ca^2+^ changes evoked by P_4_ and activates a Ca^2+^ entry through Orai1 and TRPC1, which is limited due to P_4_-dependent PGRMC1 activation. PGRMC1 downstream mechanisms involve the activation of PKA that phosphorylates Orai1 and, thus, downregulates Ca^2+^ entry and cell proliferation. (**B**) Silencing of PGRMC1 results in an increase in the P_4_-evoked STIM2-dependent Ca^2+^ entry that leads to NFAT1 translocation to the cell nucleus and, therefore, reverses the negative effect of P_4_ in the proliferation of MDA-MB-231 cells.

Figure 10 summarizes the main ideas supported by our experimental data, and according to the experimental evidence shown in the present manuscript, patients with TNBC might get the benefit of P_4_ administration if an elevated PGRMC1 expression is previously confirmed in their breast tissues samples. Although future investigation will be required to confirm this hypothesis, this idea is reinforced by the observations done in mice where P_4_ reduces tumor size and brain metastasis evoked by exogenously injected MDA-MB-231 cells [5,8,54]. Although MDA-MB-231 cells present a truncated nPR with the ability of binding P_4_, the reported effects of this hormone should be limited to the presence of alternative receptors to the classic nPR [23]. In fact, P_4_ enhances proliferation in T47-D, HER2-overexpressing cells, MCF7, and MCF10A cell lines, exhibiting normal to high nPR expression [78,79]. As previously mentioned, this discrepancy in the P_4_ effects among these cell types and the TNBCs can be explained by the different expression of the classic progesterone receptors, and particularly, by the absence or presence of the nPR, which should be considered in future investigations.

## 4. Materials and Methods

### 4.1. Material

Fura-2/AM and anti-STIM2 antibody were from ThermoFisher Scientific (Molecular Probes^®^, Madrid, Spain). PKA antagonist, KT5720, was from Abcam^®^ (Cambridge, UK). DharmaFECT Kb transfection reagent was from Dharmacon Inc. (Lafayette, CO, USA). The anti-STIM1 antibody was from BD-bioscience^®^ (Madrid, Spain). The anti-PGRMC1 antibody was from Santa Cruz Biotechnology Inc. (Dallas, USA). Polyclonal anti-VASP (Ser239) antibody was from Cell Signaling Technology^®^ (Danvers, MA, USA). HRP-conjugated secondary antibodies were from Jackson Immunoresearch Europe Ltd. (Cambridge, UK). YFP-NFAT1-reporter overexpression plasmid was kindly provided by Christoph Romanin (Johannes Kepler Institute of Biophysic, University of Linz, Linz, Austria). pCMV GEM-CEPIA1er was a gift from Masamitsu Iino (Addgene plasmid #58215; http://n2t.net/addgene:58215; RRID:Addgene_58215) [65]. BrdU cell proliferation assay kit was from BioVision Inc. (Milpitas, CA, USA). shPGRMC1 was from OriGene Technologies Inc. (Rockville, MD, USA). shOrai1 was kindly provided by Mohamed Trebak (Department of Cellular and Molecular Physiology, The Pennsylvania State University College of Medicine, Hershey, PA 17033, USA). shTRPC1 plasmid was kindly provided by Geoffrey E. Woodard (Department of Surgery, Uniformed Services University of the Health Sciences, Bethesda, MD, USA). siRNA was from Santacruz biotechnology Inc. (Heidelberg, Germany). esiSTIM1, esiPGRMC1, and esiSTIM2, thapsigargin (TG), progesterone (P_4_), 2-Aminoethoxydiphenyl borate (2-APB), anti-β-actin antibody, and other reagents of analytical grade were ordered from Sigma-Aldrich^®^ (Madrid, Spain).

### 4.2. Cell Lines and Genetic Modification

MCF10A cell line was provided by Potier-Cartereau (Université François Rabelais, Tours, France). MCF7 and the triple-negative breast cancer cell lines—MDA-MB-231, MDA-MB-468, and BT20, were collected from ATCC^®^ (Manassas, VA, USA). Cells were cultured following the manufacturer’s instructions or the protocols described elsewhere [25]. MCF7 and the TNBC cells were cultured at 37 °C with 5% CO_2_ in DMEM medium supplemented with FBS and penicillin/streptomycin; meanwhile, MCF10A cells were cultured in DMEM F-12 supplemented with horse serum, insulin, hydrocortisone, EGF, cholera toxin, and penicillin/streptomycin. Cell transfection was achieved by cell permeabilization with DharmaFECT transfection reagent, and subsequently, cells were incubated for 48 h either with 1–3 μg/mL of esiRNAs against STIM1 and STIM2 or siRNA. Alternatively, we used 2 μg/mL of scrambled plasmid, shPGRMC1, shOrai1, and shTRPC1 plasmids. In order to ascertain the efficiency of silencing protocols, Western blotting using the specific antibodies was done, as described below. Finally, cell transfection was done using the YFP-NFAT1-reporter overexpression plasmid (2 μg/mL), which was evaluated upon 72 h of transfection in order to ascertain NFAT1 translocation to the nucleus due to Ca^2+^ entry activation [80].

### 4.3. Cell Proliferation Assay

Non-genetically modified MDA-MB-231 cells (scrambled cells) or MDA-MB-231 cells transfected with the respective genetic agents were seeded at 5 × 10^5^ cells/mL in 96-well plates. Following this, cells were cultured for 72 h either in the absence (control) or presence of P_4_ (1 μM). Proliferation cell rate at time 0, 24, 48, and 72 h was analyzed by using a commercial BrdU-based proliferation kit from Abcam [25]. Briefly, cells were incubated at the time indicated with BrdU for 2 h and subsequently fixed. Upon cell permeabilization, samples were incubated with an anti-BrdU antibody and with the reporter dye for 30 min. Immediately after the addition of the stop solution, the light emitted at 650 nm by the reporter dye was monitored by using a Plate-reader (Epoch, Bioteck^®^; Swindon, UK).

### 4.4. Analysis of the Changes in the Cytosolic Free Ca^2+^ Concentration (Ca^2+^)_c_

MDA-MB-231 cells were either transfected for 48 h with the indicated genetic material or were kept untreated. The day before the experiments, cells were detached, the medium was replaced, and finally, they were seeded again at 1 × 10^5^ cells/mL in a 6-well plate containing a sterile coverslip that was deposited at the bottom of the wells. Cells were allowed to spread onto the coverslips for an additional 24 h. The next day, cells were incubated for 30 min with 2 μM of fura-2/AM at 37 °C in the incubation chamber.

Coverslips were mounted in a perfusion chamber and were maintained in a Ca^2+^-free HBS medium (in mM: 125 NaCl, 25 HEPES, 5 KCl, 1 MgCl_2_, pH 7.4, and supplemented with 0.1% (*w*/*v*) bovine serum albumin and 5 mM of D-glucose). Fluorescence emitted by the samples at 505 nm, as a result of the alternative excitation at 340/380 nm wavelengths, was recorded using a cooled sCMOS camera (Zyla 4.2, Andor, Belfast, UK). Fura-2 fluorescence was represented as the changes in the ratio of fluorescence (F_n_/F_0_) upon normalizing the ratio by considering the basal values on each experiment, which result from removing the extracellular free Ca^2+^ by supplementation of the medium with 75 μM of EGTA (F_0_). To compare among different experimental conditions, the areas under the curves were calculated by using the integral of the rise in the ratio of fura-2 fluorescence for 2–4 min after the addition of the stimuli to the cells. Ca^2+^ entry evoked by emptying the intracellular Ca^2+^ stores was visualized upon the addition of 1 mM of CaCl_2_ to the extracellular medium, as widely reported elsewhere [25].

Additionally, Ca^2+^ mobilization from the endoplasmic reticulum was determined by using the endoplasmic reticulum Ca^2+^ dye, GEM-CEPIA1er, which can be excited at 380 nm and presents two maximum emission wavelengths: at 510 nm in the absence of Ca^2+^ (green) and at 462 nm in the presence of Ca^2+^ (blue). Therefore, the ratio between both emission wavelength (blue/green) can be used to determine the intraluminal Ca^2+^ changes within the endoplasmic reticulum, as previously reported by others [65].

### 4.5. Quantification of Cell Death

MDA-MB-231 cells were transfected with shPGRMC1 or the scrambled plasmids, as aforementioned. Cells were cultured for 24 h in the absence or presence of P_4_ (1 μM) and, subsequently, they were loaded with propidium iodide (4 μM) for 45 min at 37 °C inside the incubator. Cell middle plane images were acquired at 555/624 of wavelength (Ex/Em, respectively) using a cooled digital CMOS camera (Zyla 4.2, Andor, Belfast, UK) and a fluorescence 40x objective mounted in an inverted microscope (Nikon Eclipse Ti-2, Amsterdam, The Netherlands) that is controlled by the NIS-Elements AR software (Nikon, Amsterdam, The Netherlands). Cell death was expressed as a percentage of positive PI cells with respect to the total cell account in each field.

### 4.6. Visualization of NFAT1 Translocation to the Nucleus

MDA-MB-231 cells (1 × 10^5^ cells/mL) were transfected for 48 h with the YFP-NFAT1-reporter overexpression plasmid and shPGRMC1 or with the NFAT1-reporter plasmid alone. Upon confirmation of the positive expression of NFAT1, which was done by visualizing the YFP fluorescence derived from the NFAT1-reporter in control proliferating cells, they were seeded at the appropriate concentration (1 × 10^5^ cells/mL). The following day, cells were stimulated for 48 h with P_4_ (1 μM) and were maintained growing under regular culture conditions (Mock). Alternatively, as an internal control, we artificially forced the internalization of the YFP-NFAT1-reporter into the nuclei by stimulating the cells for 24 h with 1 μM of thapsigargin (TG). This maneuver should activate SOCE and, subsequently, facilitate downstream NFAT1 constant translocation to the nucleus [69]. The analysis of the fluorescence emitted by the NFAT1-reporter was done by using an inverted fluorescence microscope at 24 h and 48 h after the beginning of the cell stimulation with P_4_. Data were normalized by comparing with the NFAT1 fluorescence with respect to the one emitted by the mock cells growing under regular conditions.

### 4.7. Western Blotting

MCF10A, MCF7, and MDA-MB-231 cells (1 × 10^6^ cells/mL) were lysed by mixing with an equal volume of NP40 buffer. The protein quantification was done using the BCA-based protocol, and all samples were normalized to 1 mg/mL, before proteins were denatured by mixing with an equal volume of Laemmli’s buffer (supplemented with SDS, 5%), following which, they were heated at 95 °C for 5 min. Proteins were separated by SDS-PAGE and were subsequently transferred to nitrocellulose membranes. Non-specific binding sites were blocked by incubating the membranes with the blocking buffer (10 % of BSA). Western blotting was done by incubating the membranes overnight either with the specific monoclonal anti-STIM1, anti-STIM2, anti-PGRMC1, and anti-VASP antibodies diluted in TBST (supplemented with BSA at 1:1000). The excess of the primary antibody was removed by washing for 30 min with TBST. Following this, the membranes were incubated for an additional hour with the appropriate HRP-conjugated secondary antibodies (diluted 1:10,000 in TBST). Finally, the membranes were exposed to the dura-ECL solution for 5 min. Chemiluminescence images were acquired using a CD-digit chemiluminescence device (Licor^®^, Bonsai Lab, Madrid, Spain), and optic densitometry of the images was analyzed by using the Image J (Fiji plugging) (free-software from NIH). Membranes were reprobed with anti-β-actin antibody as a loading control.

### 4.8. Statistical Analysis

A student’s *t*-test was used for establishing a comparison between two groups. Meanwhile, for multiple comparisons, the analysis of the variance (ANOVA), combined with post hoc Tukey’s test and Dunnett’s tests, was used. *p* < 0.05 was considered a significant difference between groups.

## 5. Conclusions

1. P_4_ activates a STIM2-dependent Ca^2+^ entry that occurs through Orai1 and TRPC1 in MDA-MB-231 cells.
2. PGRMC1 activates PKA, which phosphorylates Orai1 to downregulate the P_4_-evoked Ca^2+^ entry.
3. P_4_-evoked PGRMC1 activation downregulates cell proliferation in MDA-MB-231 cells.
4. The silencing of PGRMC1 impairs P_4_-evoked cell death in MDA-MB-231 cells.
5. The silencing of PGRMC1 results in an elevated Ca^2+^ entry and, subsequently, increases NFAT1 translocation to the cell nucleus.
6. PGRMC1 could be targeted to prevent uncontrolled cell proliferation in TNBC.

## Abbreviations

2-APB	2-aminoethoxydiphenyl borate
cAMP	3′,5′-cyclic adenosine monophosphate
GEM-CEPIA1er	Endoplasmic reticulum intraluminal Ca^2+^ dye
IP_3_R	Inositol 1,4,5-trisphosphate receptor
mPRα	Progesterone receptor membrane α
NFAT1	Nuclear factor of activated T-cells 1
nPR	Nuclear progesterone receptor
P_4_	Progesterone
PGRMC1	Progesterone receptor membrane component 1
PKAi	PKA antagonist, KT5720
SERCA	Sarcoendoplamic reticulum Ca^2+^-ATPase
SOCE	Store-operated calcium entry
STIM1 & 2	Stromal interaction molecule 1 & 2
TG	Thapsigargin
TNBC	Triple negative breast cancer cells
EGTA	Ethylene glycol-bis(β-aminoethyl ether)-N,N,N′,N′-tetraacetic acid
